# Supplementary figures and images for: Integration of lncRNA–miRNA–mRNA reveals novel insights into oviposition regulation in honey bees
Source: PeerJ. 2017 Oct 5;5:e3881. doi: 10.7717/peerj.3881 (PMC5632538; doi:10.7717/peerj.3881)

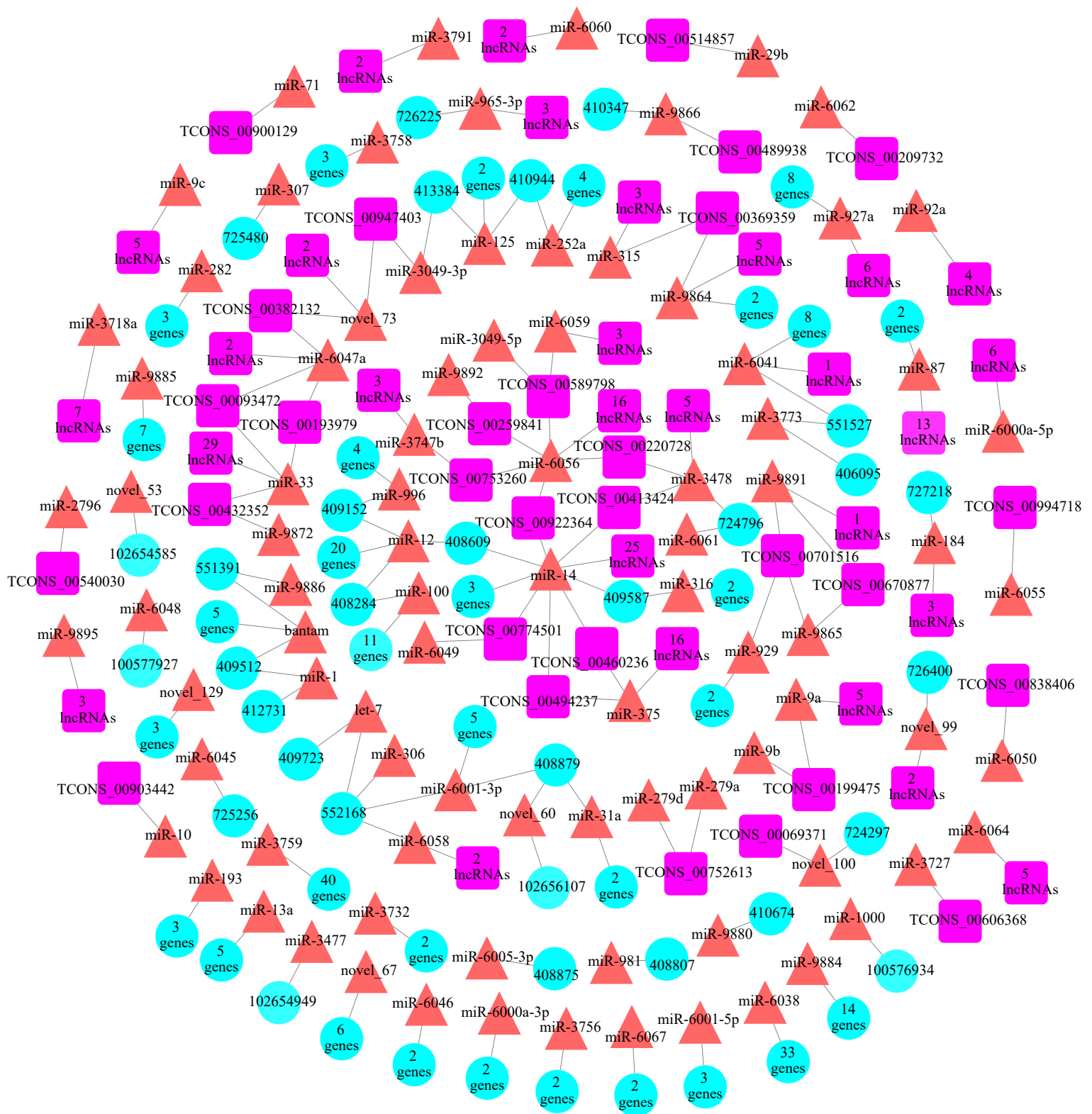

Supplement: Figure S1 — Purple square nodes represent lncRNAs. Red triangle nodes represent miRNAs. Blue circle nodes represent mRNAs. [file peerj-05-3881-s009.pdf]
